# Computer-Assisted CBCT Evaluation of Inferior Alveolar Nerve Canal Regeneration One Year Following Nerve Transposition

**DOI:** 10.3390/jcm15030985

**Published:** 2026-01-26

**Authors:** Fares Kablan, Shadi Daoud, Amjad Shhadeh, Samer Srouji

**Affiliations:** 1 Department of Oral and Maxillofacial Surgery, Galilee College of Dental Sciences, Galilee Medical Center, Nahariya 2210001, Israel; 2 The Azrieli Faculty of Medicine, Bar-Ilan University, Safed 1311502, Israel

**Keywords:** mandibular canal, inferior alveolar nerve, nerve transposition, mandibular canal regeneration, computer-assisted analysis

## Abstract

**Background**: Rehabilitation of the severely atrophic posterior mandible remains surgically challenging, and inferior alveolar nerve (IAN) repositioning is a well-established technique that enables implant placement in anatomically compromised cases. Although neurosensory outcomes following nerve relocation have been extensively investigated, the regenerative capacity of the mandibular canal itself has not been previously evaluated. This study presents the first computer-assisted, cone-beam computed tomography (CBCT)-based assessment of bony canal regeneration after IAN transposition. **Methods**: Twenty-two patients who underwent unilateral IAN transposition were evaluated using standardized CBCT one year postoperatively. A semi-manual segmentation workflow was performed using Mimics Core Medical software version 27.0 (Materialise), and regenerated canal walls were identified according to four strict criteria: (1) canal continuity across sequential CBCT sections, (2) defined canal walls demonstrating high-density bone (>800 HU, or >400 HU), (3) ≥270° circumferential bony enclosure, and (4) morphology consistent with the native mandibular canal. Regeneration was quantified as the proportion of the surgically disrupted canal segment exhibiting a fully, or near fully, reconstructed canal. **Results**: Mandibular canal regeneration was observed in all patients. The mean regeneration at one year was 72.7% ± 13% when applying strict >800 HU criteria, with 20 patients demonstrating substantial (>70%) reformation and 2 patients showing partial regeneration (<40%). When a lower density threshold (>400 HU) was applied to include early or less mineralized bone, the mean regeneration increased to 78.1% ± 11%, indicating the presence of maturing bone structures that did not yet meet full-density criteria. **Conclusions**: Computer-assisted CBCT analysis demonstrates that partial to extensive regeneration of the mandibular canal occurs within one year following IAN transposition. This study provides the first quantitative evidence of this phenomenon, highlighting the intrinsic regenerative potential of the mandibular canal and suggesting a possible association with postoperative neurosensory recovery.

## 1. Introduction

Management of the severely atrophic posterior mandible requires advanced surgical strategies due to the marked loss of vertical bone height and the proximity of the inferior alveolar nerve (IAN) [1,2]. When residual bone is insufficient for the placement of standard-length implants, inferior alveolar nerve repositioning, via lateralization or transposition, offers a predictable solution that permits implant anchorage in native basal bone while reducing the risk of direct nerve injury [3]. Since first described by Jensen and Nock in 1987 [4], IAN repositioning has evolved into an established technique with well-documented clinical outcomes, enabling implant placement even in cases with only 3–4 mm of bone above the mandibular canal [5,6,7].

Two principal approaches are used. IAN lateralization preserves the continuity of the mental nerve by gently retracting the neurovascular bundle laterally through a corticotomy window in the lateral mandibular cortex. IAN transposition, in contrast, involves a more extensive corticotomy with intentional sacrifice of the incisive branch to mobilize the mental foramen posteriorly and allow broader implant access. Both techniques have demonstrated high success rates and function reliably when properly executed [5,6,7,8,9].

Neurosensory outcomes following IAN repositioning are well characterized. Transient postoperative hypoesthesia or paresthesia is common, but systematic reviews consistently demonstrate high rates of sensory recovery within 6–12 months when a meticulous surgical technique is employed [10,11,12,13]. Implant survival and long-term peri-implant stability similarly approach outcomes achieved in native bone, with reported survival rates of approximately 90% and stable bone levels over extended follow-up [6,7,10,11,14].

However, while surgical protocols, neurosensory sequelae, and implant-related outcomes have been widely studied, one critical anatomical consequence of IAN repositioning remains virtually unexplored: the fate of the mandibular canal itself. Because the mandibular canal is necessarily disrupted during lateralization or transposition, the repositioned nerve lies temporarily without its natural bony housing. Whether this canal regenerates—and if so, to what extent, over what timeline, and with what morphological characteristics—has not been systematically investigated in humans. To date, only two case reports have described radiographic evidence of canal wall reformation years after IAN repositioning [15,16], leaving the biological behavior of the mandibular canal following transposition or lateralization largely unknown.

Animal studies provide compelling evidence that such regeneration is biologically plausible. Models in rabbits, canines, and rodents demonstrate that peripheral nerves can promote local osteogenesis through nerve-dependent signaling pathways, including NGF-mediated osteoid formation and transcriptional regulation of osteogenic markers such as RUNX2 and osteopontin [17]. Micro-CT studies have also shown reconstitution of the mandibular canal during bone regeneration phases in distraction osteogenesis models [18]. These findings align with embryological principles: the mandibular canal forms around the developing IAN rather than the nerve growing into a pre-existing canal, suggesting an inherent biological capacity for canal formation around a nerve.

The clinical significance of this phenomenon is substantial. Exposed peripheral nerves lacking bony protection may be more susceptible to mechanical irritation and hypersensitivity. Conversely, re-establishment of a protective bony canal may contribute to sensory normalization after nerve repositioning [10,19]. Understanding canal regeneration is therefore relevant not only to IAN lateralization and transposition, but also to other procedures in which the canal is disrupted, including sagittal split osteotomy, mandibular reconstruction, and vertical ridge augmentation [20,21].

Despite its clinical relevance and biological plausibility, mandibular canal regeneration following inferior alveolar nerve transposition or lateralization has not been systematically evaluated in humans. The present study therefore aimed to assess mandibular canal regeneration one year after inferior alveolar nerve transposition using computer-assisted cone-beam computed tomography (CBCT) analysis, focusing on canal formation, canal continuity, and morphological characteristics in severely atrophic mandibles.

## 2. Materials and Methods

### 2.1. Study Design and Participants

This retrospective cohort study was conducted at the Department of Oral and Maxillofacial Surgery, Galilee Medical Center, between 2019 and 2024. During this period, 117 patients underwent IAN repositioning procedures. From this larger cohort, only 22 patients fulfilled the strict inclusion criteria required for this radiographic investigation. Eligible patients had completed a full one-year clinical and radiographic follow-up after unilateral IAN transposition, exhibited no postoperative complications such as infection, wound dehiscence, or implant failure, and had no history of systemic bone disease or metabolic disorders known to affect bone regeneration. Only sites reconstructed with allograft alone—without autogenous or xenograft block grafts—were included to eliminate confounding variability in bone healing dynamics. High-quality CBCT scans suitable for three-dimensional evaluation were required. Data for this study were collected between September 2024 and April 2025, ensuring that all included patients completed the standardized one-year postoperative CBCT within this period. The study was approved by the Institutional Review Board of Galilee Medical Center and conducted in accordance with the Declaration of Helsinki (Protocol code 0130-24-NHR, 20 July 2024).

### 2.2. Surgical Protocol

All surgical procedures were performed under general anesthesia following established protocols for IAN repositioning. After elevation of a full-thickness mucoperiosteal flap, the lateral mandibular cortex was exposed and a corticotomy window was created using rotary or piezoelectric instruments (Piezosurgery+, Mectron, Carasco, Italy). This cortical window was temporarily removed to expose the neurovascular bundle. The IAN was gently mobilized with blunt dissection and retracted laterally to allow placement of endosseous implants in the basal bone. The incisive branch was intentionally transected to permit posterior repositioning of the mental foramen when clinically indicated. After implant placement, the cortical window was either repositioned or particulated and placed over the nerve, and allograft particulate bone combined with a resorbable membrane was applied to prevent direct nerve–implant contact and enhance healing. The mucoperiosteal flap was then repositioned and closed without tension [3,22]. All procedures were performed by a single experienced surgeon (Dr. Fares Kablan), thereby minimizing technical variability across the cohort [13,23].

### 2.3. Imaging Protocol

CBCT imaging was performed one year postoperatively to evaluate mandibular canal regeneration. All scans were acquired using the Planmeca ProMax^®^ 3D Classic (Planmeca Oy, Helsinki, Finland) under standardized conditions, including a voxel size of 0.3 mm, tube voltage of 106 kV, tube current of approximately 65 mAs, an 8 × 8 cm field of view, and a scan time of 12 s. Patients were positioned with the Frankfurt plane parallel to the floor to ensure reproducibility.

### 2.4. Segmentation Workflow

Image processing and three-dimensional reconstruction were performed using Mimics Core Medical software version 27.0 (Materialise, Leuven, Belgium). A semi-automated segmentation workflow was employed to enable accurate delineation of the mandible, implants, IAN and regenerated mandibular canal. Segmentation was guided by Hounsfield Unit (HU) thresholding: bone was identified using a range of 400–3071 HU, while regenerated canal walls were segmented using two defined thresholds, >800 HU, corresponding to mature bone, and >400 HU, representing early or partially mineralized bone undergoing maturation.

We acknowledge the known variability between CBCT-derived gray values and CT-based HU, and therefore selected a deliberately stricter HU range to ensure that our thresholds are robust, clinically meaningful, and appropriate for interpretation across both CBCT and CT modalities.

**Figure 1:**
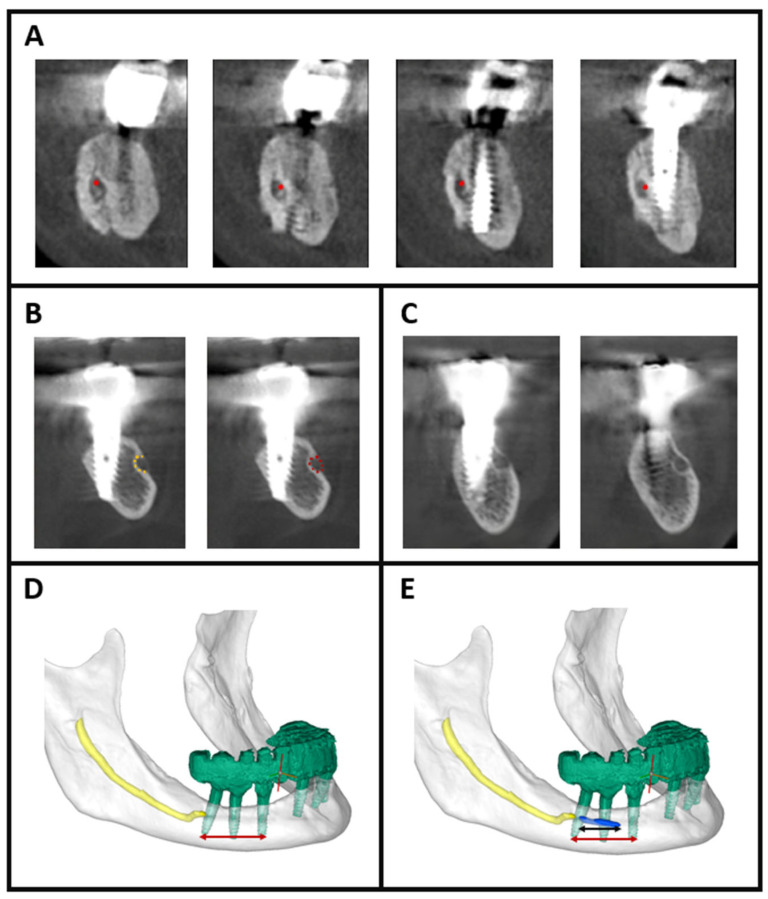
Evaluation and segmentation of mandibular canal regeneration. (**A**) Sequential cross-sectional CBCT slices demonstrating mandibular canal continuity across consecutive sections, indicated by red markers. (**B**) Example of a partially regenerated canal. When applying the strict >800 HU criterion, the yellow-marked regions exhibit adequate density but may demonstrate <270° of circumferential enclosure. When applying the >400 HU threshold, a more complete circular canal outline becomes visible, marked in red. (**C**) Cross-sectional evaluation of a region with poor canal morphology (Left slice). To ensure accurate identification and continuity assessment, the following slice (right) was reviewed, confirming the position and linear progression of the canal. (**D**) Three-dimensional segmentation showing the mandible (white, semi-transparent), implants (green), and the inferior alveolar nerve (yellow). The red linear measurement represents the defined defect length between the mesial and distal implant apices, corresponding to the surgically disrupted canal segment. (**E**) The same sagittal view with the segmented regenerated canal (blue). The black measurement line indicates the length of the regenerated canal segment used to calculate the percentage of regeneration.

Identification of canal regeneration was based on four strict and standardized criteria. First, canal continuity across sequential CBCT sections was required to confirm that the regenerated structure represented a true linear canal rather than an isolated hypodense or irregular area (Figure 1A). Second, the regenerated walls were required to demonstrate high-density bone, defined as >800 HU for mature formation or >400 HU for early-stage mineralizing bone (Figure 1B). Third, regenerated segments needed to exhibit ≥270° circumferential bony enclosure around the neurovascular bundle, indicating substantial canal reformation (Figure 1B). Finally, the reconstructed segment had to show morphological similarity to the native mandibular canal, characterized by a tubular or circular configuration in cross-sectional view (Figure 1C).

A semi-automated segmentation approach was deliberately selected to minimize inaccuracies associated with metallic implant artifacts and the irregular healing patterns commonly encountered after IAN repositioning. Following segmentation, all objects—including the mandible, implants, IAN and regenerated canal—were independently reviewed and validated by two oral and maxillofacial surgeons, each with more than ten years of CBCT interpretation experience, ensuring accuracy and minimizing interobserver variability.

### 2.5. Assessment of Canal Regeneration

To precisely evaluate canal regeneration, the portion of the mandibular canal disrupted during surgery was defined as the linear distance between the mesial apex of the mesial implant and the distal apex of the distal implant. This defined defect length represented the exact anatomical region in which canal continuity had been surgically interrupted (Figure 1D).

Regeneration was assessed on the same standardized sagittal view, which was used both to measure the defined defect length and to quantify the length of the regenerated canal. Only canal segments that fulfilled all four strict criteria were included in the measurement (Figure 1E).

The percentage of canal regeneration was calculated using the following formula:

$$\mathrm{Regeneration}\;(\%)=\left(\frac{\mathrm{Length}\;\mathrm{of}\;\mathrm{Regenerated}\;\mathrm{Canal}}{\mathrm{Defined}\;\mathrm{Defect}\;\mathrm{Length}}\right)\times 100\%$$

This approach reflects true restoration of canal continuity and architecture, rather than generalized bone fill or volumetric healing.

### 2.6. Statistical Analysis

Data were analyzed using GraphPad Prism version 8 (GraphPad Software, San Diego, CA, USA). Normality of continuous variables was assessed using the Shapiro–Wilk test. Descriptive data are presented as mean ± standard deviation.

No power calculation was performed, as this retrospective study included all eligible cases meeting the predefined inclusion criteria during the study period.

## 3. Results

**Figure 2:**
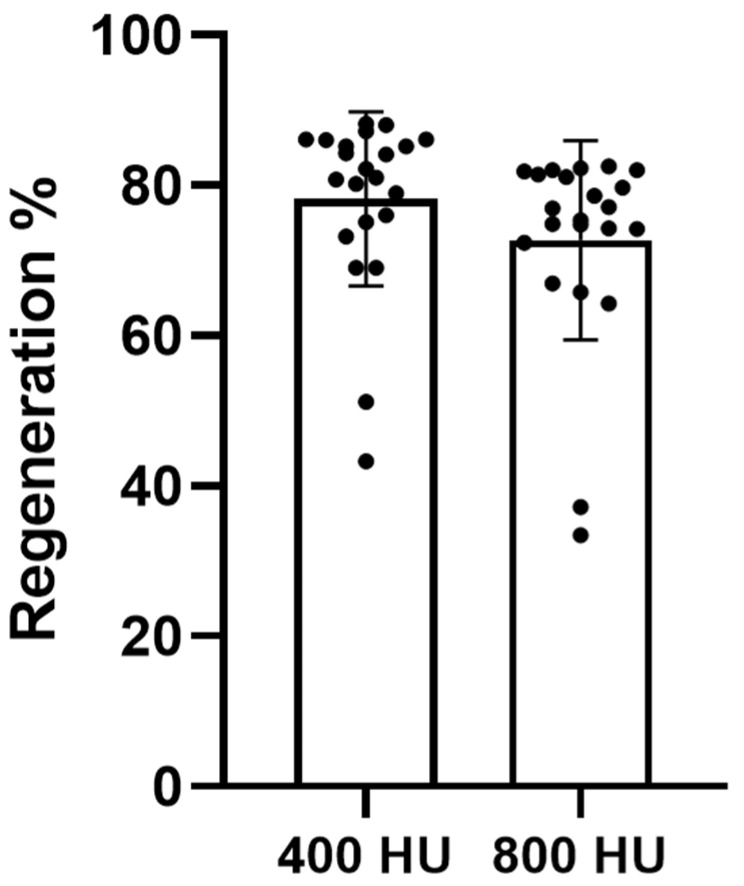
Mandibular canal regeneration at one year using 400 HU and 800 HU thresholds. Bar plots illustrate regeneration percentages calculated using two radiodensity thresholds (≥400 HU and ≥800 HU). Individual data points represent regeneration values for each of the 22 patients, overlaid on the group bars. Bars indicate mean ± SD.

Mandibular canal regeneration was consistently observed in all patients at the one-year postoperative CBCT evaluation. The mean percentage of canal regeneration in the >800 HU group, calculated based on the linear restoration of canal continuity within the defined defect segment, was 72.7% ± 13% across the cohort. Twenty patients demonstrated substantial regeneration exceeding 70%, whereas two patients exhibited partial regeneration, with restoration of <50% of the defined defect length (Figure 2).

When a lower density threshold (>400 HU) was applied to include early or partially mineralized bone, the measured extent of regeneration increased. Under this expanded threshold, the mean regeneration percentage rose to 78.1% ± 11%, reflecting the presence of less-dense but structurally relevant canal-forming bone that did not meet the strict >800 HU criteria (Figure 2).

These findings indicate that the majority of patients developed extensive canal reformation within one year following IAN transposition, and that early-stage bone maturation may underestimate true regenerative progression when using high-density thresholds alone.

## 4. Discussion

Mandibular canal regeneration following IAN transposition has remained largely unexplored, with the majority of existing literature focusing on implant success rates and neurosensory recovery [10,11,14]. Although IAN repositioning procedures, such as lateralization and transposition, are well-documented in terms of safety and reliability, the biological fate of the surgically interrupted mandibular canal has never been systematically studied [3,5,6]. This investigation offers the first computer-assisted, three-dimensional CBCT-based evaluation of canal regeneration after IAN transposition, demonstrating that canal reformation is not a rare or incidental finding but rather a consistent and quantifiable biological response.

At one year postoperatively, the mandibular canal regenerated on average by 72.7% ± 13% using a strict density threshold of >800 HU, consistent with mature bone. When a lower threshold of >400 HU was applied to include early mineralizing bone, the average regeneration increased to 78.1% ± 11%. This dual-threshold methodology was purposefully designed to differentiate between mature canal reconstitution and earlier stages of osteoid maturation. Notably, only two patients demonstrated <50% regeneration, underscoring the predominance of partial to near-complete canal restoration following transposition.

To ensure anatomical relevance, canal regeneration was defined using four standardized criteria: (1) continuity across sequential CBCT slices, (2) bony walls with density exceeding 800 HU or 400 HU depending on maturation stage, (3) ≥270° circumferential bony enclosure of the nerve, and (4) morphology consistent with the native mandibular canal. These criteria were critical to exclude incidental radiopacities and confirm true canal-like reconstruction.

The biological plausibility of canal regeneration is supported by established principles of neuro-osteogenic interaction. During embryogenesis, mandibular canal formation is driven by osteogenic signaling from the developing IAN [24]. Preclinical studies in animal models have demonstrated that peripheral nerves contribute to osteogenesis via neurotrophic mechanisms, including NGF-mediated osteoid deposition and upregulation of osteogenic markers such as RUNX2 and osteopontin [18]. Our findings suggest that similar nerve-mediated pathways may be reactivated following surgical manipulation, allowing for canal regeneration even in the absence of grafts or biological stimulants [19,20].

From a clinical standpoint, the regenerative capacity of the mandibular canal may have implications for neurosensory recovery. In our cohort, patients who exhibited greater canal regeneration were more likely to report improved or normalized sensation, while those with limited bony restoration tended to experience persistent dysesthesia or hypoesthesia. Although our study was not powered to evaluate this relationship statistically, the observed association supports the hypothesis that bony canal reformation may provide mechanical stabilization, limit perineural irritation, and preserve a favorable microenvironment—factors that collectively support neural recovery [24]. However, this observation is preliminary and cannot be interpreted as evidence; any meaningful evaluation of a potential relationship between canal regeneration and neurosensory recovery will require a dedicated prospective study employing standardized functional sensory testing.

These insights may extend beyond IAN transposition. Mandibular canal disruption is also encountered in sagittal split osteotomy and distraction osteogenesis. In these contexts, canal regeneration may represent an underrecognized element of osseous healing and could influence long-term neurosensory prognosis [20,21].

To reduce potential confounding, we limited our analysis to unilateral cases, thereby avoiding intra-patient anatomical variability. Additionally, all surgeries were performed by a single experienced operator to enhance procedural consistency. Nonetheless, this study has several limitations. We acknowledge that the retrospective design and modest sample size (*n* = 22) limit statistical power and generalizability, as only a subset of patients from the larger surgical cohort met the strict inclusion criteria required for this radiographic analysis. CBCT imaging provides detailed morphological assessment but lacks histologic validation of bone quality. Sensory outcomes were qualitatively assessed and not formally correlated with radiographic findings—an aspect under investigation in our ongoing work.

In addition, it is important to acknowledge that histological confirmation of mandibular canal remodeling is not feasible in human subjects; therefore, the present findings are limited to radiographic evidence of bony regeneration. Moreover, due to these inherent constraints, no causal relationship between canal regeneration and neurosensory recovery can be inferred from this observational study.

Future plans for this study may include conducting dedicated in vitro and in vivo investigations to clarify the potential influence of the inferior alveolar nerve on canal bone remodeling, as these models may provide the histological insight not attainable in human subjects. In parallel, we are preparing a prospective clinical study with standardized neurosensory functional testing to more rigorously evaluate the relationship between canal remodeling and sensory recovery.

## 5. Conclusions

This study provides the first structured, quantitative evidence that the mandibular canal possesses intrinsic regenerative capacity following surgical disruption. Most patients demonstrated substantial reformation of the canal within the first postoperative year. These findings challenge long-standing assumptions about the irreversibility of canal removal and open new avenues for understanding mandibular canal healing biology. Future research should aim to track the longitudinal progression of canal maturation, identify biologic or surgical factors that influence regeneration, and clarify its role in facilitating neurosensory recovery.

## Data Availability

The data supporting the findings of this study are available from the Department of Oral and Maxillofacial Surgery, Galilee Medical Center, in anonymized form and can be made available by the corresponding author upon reasonable request, subject to institutional and ethical approval.

## Abbreviations

The following abbreviations are used in this manuscript:

IAN	Inferior Alveolar Nerve
CBCT	Cone-Beam Computed Tomography
HU	Hounsfield Units
NGF	Nerve Growth Factor
RUNX2	Runt-Related Transcription Factor 2
